# Real-World Safety and Efficacy of Nivolumab in Advanced Squamous and Nonsquamous Non-Small-Cell Lung Cancer: A Retrospective Cohort Study in Croatia, Hungary, and Malta

**DOI:** 10.1155/2020/9246758

**Published:** 2020-11-29

**Authors:** Eduard Vrdoljak, Marko Jakopović, Lajos Geczi, Krisztina Bogos, Lidija Bošković, Claude Magri, Lela Bitar, Žarko Bajić, Miroslav Samaržija

**Affiliations:** ^1^Department of Oncology, Clinical Hospital Center Split, School of Medicine, University of Split, Split, Croatia; ^2^Department for Respiratory Disease “Jordanovac”, University Hospital Center Zagreb, Zagreb, Croatia; ^3^School of Medicine, University of Zagreb, Zagreb, Croatia; ^4^National Institute of Oncology, Budapest, Hungary; ^5^Department of Pulmonology, National Korányi Institute of Pulmonology, Budapest, Hungary; ^6^Mater Dei Hospital, Triq Dun Karm, L-Imsida, Malta; ^7^Scientific Unit “Dr. Mirko Grmek”, Psychiatric Hospital “Sveti Ivan”, Zagreb, Croatia

## Abstract

**Background:**

There is a lack of real-world data on the safety and efficacy of nivolumab in patients with previously treated advanced non-small-cell lung cancer (NSCLC) especially in South East Europe, a region with particularly high incidence and an unfavorable mortality-to-incidence ratio for lung cancer.

**Objectives:**

To evaluate the real-world safety and efficacy of nivolumab in patients with previously treated advanced squamous and nonsquamous NSCLC in South East Europe.

**Methods:**

This is a multicenter, retrospective cohort study on patients with stage IIIB or IV disease with at least one previous systemic treatment who received nivolumab through an expanded-access program between 2015 and 2017 in Croatia, Malta, and Hungary. The primary endpoint was the proportion of patients whose therapy was discontinued because of toxicity. Secondary endpoints were the incidence of adverse events (AEs), objective response rate (ORR), disease control rate (DCR), time to response (TTR), duration of response (DOR), progression-free survival (PFS), and overall survival (OS).

**Results:**

We analyzed data on 239 patients with a median (IQR) age of 62 (57–68), and 33% of them were women. Treatment was discontinued because of toxicity in 11.6% (95% CI 7.8% to 16.5%) of patients. The PFS was 6.4 (95% CI 5.2 to 8.6) months, and the median OS was 14.1 (10.6 to 18.0) months.

**Conclusions:**

The safety and efficacy of nivolumab in previously treated patients with advanced NSCLC in the real-world South East Europe clinical settings were consistent with the results of randomized clinical trials and comparable to the results from other countries.

## 1. Introduction

Despite the accelerated lowering of lung cancer mortality rates in the USA during the last decade, it still causes more deaths than breast, prostate, colorectal, and brain cancers together [1]. Similarly, lung cancer is responsible for 18% of all cancer-related deaths worldwide, and it is still the most often diagnosed cancer [2]. Its age-standardized rates are particularly high in Central and Eastern Europe, with the age-standardized rate in Hungary as high as 77.4/100.000 males in 2018 [3]. Approximately, 80% of all diagnosed lung cancers are non-small-cell carcinoma, histologically further classified into two predominant groups: squamous cell NSCLC in approximately 25% of cases and nonsquamous cells, primarily adenocarcinoma, in 45% of cases [4]. Treatment options for patients with non-small-cell lung carcinoma (NSCLC) after the failure of first-line platinum-based chemotherapy are limited. The recommended second-line treatment of patients with advanced NSCLC without actionable mutations has, until recently, mainly included docetaxel and pemetrexed (only for cases with a nonsquamous histology) [5]. Pemetrexed demonstrated a similar clinical response as docetaxel, but a better safety profile. Both agents provided a median survival of approximately 7–8 months and a response rate <10%, with less hematological toxicity in the pemetrexed arm [5, 6]. In 2014, the first immunotherapy as a second-line treatment for NSCLC became available, and nivolumab, a fully human IgG4 monoclonal antibody that binds to the programmed death 1 (PD-1) immune checkpoint and inhibits its interaction with ligands PD-L1 and PD-L2 [7], was shown to be markedly more effective than docetaxel in advanced squamous [8] and nonsquamous NSCLC [9], with a duration of response of 23.8 months with nivolumab vs. 5.6 months with docetaxel [10] and an estimated 5-year OS rate of 16% of both agents in both squamous and nonsquamous NSCLC [11].

Randomized controlled clinical trials often have lower generalizability due to their narrower targeted populations, restrictive inclusion, and exclusion criteria, precisely controlled concomitant therapy, and strictly defined and followed procedures. They often have a less comprehensive assessment of more rare adverse events due to their smaller sample sizes and selected participants. They are often highly homogenous, with less than 1% of cancer patients being from South East Europe [12]. Therefore, is it important to evaluate randomized clinical trial results in real-world, postmarketing setting. This was the aim of our study. Our primary objective was to evaluate the real-world safety and tolerability of nivolumab in NSCLC that progressed or recurred during or after at least one line of systemic therapy for advanced or metastatic disease in South East Europe. The secondary objective was to assess the real-world efficacy of nivolumab in the same population. We also planned to compare patients with squamous NSCLC with patients with nonsquamous NSCLC and those with CNS metastasis, who are often excluded from phase II and III clinical trials.

## 2. Methods

### 2.1. Study Design

We performed this multicenter, retrospective, observational study with real-world clinical data from a cohort of patients who received nivolumab under an expanded-access program from June 17, 2015, to October 31, 2017, at the Department of Oncology, Clinical Hospital Center Split, Split, Croatia, the Department for Respiratory Disease “Jordanovac,” University Hospital Center Zagreb, Zagreb, Croatia, the Department of Pulmonology, National Korányi Institute of Pulmonology, Budapest, Hungary, and Mater Dei Hospital, Triq Dun Karm, L-Imsida, Malta. The ethics committees of the participating institutions approved the study protocol, and we obtained written informed consent from all living patients. The data were anonymized before the analysis. The study was conducted in accordance with the World Medical Association Declaration of Helsinki of 1975 as revised in 2013 [13] and the International Conference on Harmonization Guidelines on Good Clinical Practice. The study protocol was not preregistered in any public registry. The study was sponsored by Bristol-Myers Squibb.

### 2.2. Study Population

Inclusion criteria for enrollment in the study were age ≥18 years, histologically or cytologically confirmed squamous or nonsquamous NSCLC patients who presented with stage IIIB or stage IV disease (according to version 7 of the International Association for the Study of Lung Cancer Staging Manual in Thoracic Oncology) or with recurrent or progressive disease following multimodal therapy (radiation therapy, surgical resection, or definitive chemoradiation therapy for locally advanced disease), and patients with an Eastern Cooperative Oncology Group (ECOG) performance status (PS) of ≤2 and who experienced disease progression or recurrence during or after at least one systemic therapy for advanced or metastatic disease. Patients who received platinum-containing adjuvant, neoadjuvant, or definitive chemoradiation therapy for locally advanced disease and developed recurrent (local or metastatic) disease within 6 months of completing therapy were eligible. Subjects with recurrent disease >6 months after completing platinum-containing adjuvant, neoadjuvant, or definitive chemoradiation therapy for locally advanced disease who also subsequently progressed during or after a systemic regimen given to treat the recurrence were eligible. Prior chemotherapy, TKI, or immunotherapy (tumor vaccines, cytokines, or growth factors given to control cancer) must have been completed at least 2 weeks before the program drug administration, and all adverse events had to have either resolved or stabilized. Patients were eligible if CNS metastases were treated and patients had neurologically returned to baseline (except for residual signs or symptoms related to the CNS treatment) and remained there for at least 14 days prior to enrollment. In addition, patients must either have been off corticosteroids or on a stable dose or decreasing dose of ≤10 mg daily prednisone (or equivalent). Patients with type I diabetes mellitus, residual hypothyroidism due to an autoimmune condition requiring hormone replacement, psoriasis not requiring systemic treatment, or conditions not expected to recur in the absence of an external trigger were permitted to enroll.

Exclusion criteria were ECOG performance status ≥3, life expectancy of <6 weeks, active brain or leptomeningeal metastasis, carcinomatous meningitis, ocular melanoma, known or suspected autoimmune disease, prior therapy with anti-PD-1, anti-PD-L1, anti-PD-L2, anti-CT137, or anti-CTLA-4 antibodies including ipilimumab or any other antibody or drug specifically targeting T cell costimulation or checkpoint pathways, prior treatment in any nivolumab trial including prior treatment in either arms of the nivolumab studies CA209057 or CA209026, interstitial lung disease that was symptomatic or might have interfered with the detection or management of suspected drug-related pulmonary toxicity, other active malignancy requiring concurrent intervention, known alcohol or drug abuse, known history of testing positive for human immunodeficiency virus (HIV) or known acquired immunodeficiency syndrome (AIDS), history of severe hypersensitivity reactions to other monoclonal antibodies, history of allergy or intolerance to program drug components or polysorbate-80-containing infusions, pregnancy and breastfeeding, and prior malignancy active within the previous three years except for locally curable cancers that had been apparently cured, such as basal or squamous cell skin cancer, superficial bladder cancer, or carcinoma in situ of the prostate, cervix, or breast.

Screening laboratory values must have met the following criteria prior to commencement of treatment: WBCs ≥ 2000/*µ*L, neutrophils ≥ 1500/*µ*L, platelets ≥ 100 × 10³/*µ*L, hemoglobin ≥ 9.0 g/dL, serum creatinine of ≤ 1.5 × ULN or creatinine clearance (CrCl) > 40 mL/minute (using Cockcroft/Gault formula), female: CrCl = [(140 − age in years) × weight in kg × 0.85)/(72 × serum creatinine in mg/dL)], male: CrCl = [(140 − age in years) × weight in kg × 1.00)/(72 × serum creatinine in mg/dL)], AST ≤ 3 × ULN, ALT ≤ 3 × ULN, and total bilirubin ≤ 1.5 × ULN (except patients with Gilbert Syndrome, who must have had total bilirubin <3.0 mg/dL).

### 2.3. Sample Type and Required Sample Size

We analyzed data on the entire population and not only data on the sample of patients treated in the participating institutions during the study period. The power analysis was not performed prior to the beginning of data collection.

### 2.4. Endpoints

The primary endpoint was the proportion of patients receiving at least one dose of nivolumab whose treatment with nivolumab was discontinued because of toxicity. Secondary safety endpoints were the incidence of treatment-related adverse events of any grade, the incidence of grade III or IV adverse events according to Common Terminology Criteria for Adverse Events v4.0, and the incidence of any treatment-related immune-mediated adverse events in patients who received at least one dose of nivolumab. Secondary efficacy endpoints were the best objective response; the objective response rate (ORR), which included partial and complete response according to the response evaluation criteria in solid tumors (RECIST); the disease control rate (DCR), defined as a partial or complete response or stable disease; the time to response (TTR), defined as the time in months from the introduction of nivolumab to the date of first documented complete or partial response; the duration of response (DOR), defined for responder patients as the time in months from the date of the best response to the date of progression; the progression-free survival (PFS), defined as the time in months from the initiation of treatment with nivolumab to the date of progression or death from any cause; and the overall survival (OS), defined as the time in months from the initiation of nivolumab to the date of death from any cause. DOR data from responder patients with no progression were censored at the time of their last visit prior to the first subsequent therapy or death. PFS data from living patients without tumor progression were censored at the time of their last visit prior to the first subsequent therapy. OS data from living patients were censored at the time of their last visit.

### 2.5. Treatment

Treatment with nivolumab (Opdivo, Bristol-Myers Squibb) monotherapy was given within the named patient program (NPP) and in everyday clinical practice. Within the NPP, nivolumab was administered at a dosage of 3 mg/kg by 60-minute intravenous infusion every two weeks to a maximum of 24 months or until unacceptable toxicity and disease progression of withdrawal of informed consent.

### 2.6. Other Variables

Potential confounders whose effects we tried to control by using them as covariates in the multivariable analysis were age at the introduction of nivolumab, sex, previous treatment of metastatic disease with tyrosine kinase inhibitors, number of metastatic sites, ECOG performance status at introduction of nivolumab, and time from diagnosis to the introduction of nivolumab (years), as well as, as a time-dependent covariate, concomitant radiotherapy. We planned to control for EGFR status and anaplastic lymphoma kinase (ALK) rearrangements as covariates, but we did not include them in the multivariable analysis because of the large number of patients with unknown status. In the analysis of OS, in addition to the abovementioned covariates, we controlled the potential confounding effect of the therapy after the nivolumab and as a time-dependent covariate, the number of nivolumab cycles. Other variables that we used to describe the samples from the targeted population and treatment were age at diagnosis; stage at the introduction of nivolumab; previous chemotherapy treatment of metastatic disease; particular metastatic sites; EGFR status; ALK rearrangements; duration of treatment with nivolumab, which was not included in the multivariable analysis to avoid multicollinearity with the number of treatment cycles; and concomitant therapy with antihypertensives, proton pump inhibitors, antibiotics, corticosteroids, or acetylsalicylic acid. We obtained the data from the participating hospital electronic medical records.

### 2.7. Statistical Analysis

We used the Kaplan–Meier method to estimate the median TTR, DOR, PFS, and OS with 95% confidence intervals (CIs) and used the two-sided long-rank test to test the significance of the differences between squamous and nonsquamous NSCLC without adjustments for planned covariates. We used a Cox proportional hazards regression model with the Efron method to handle ties to estimate the hazard ratios (HRs) for the efficacy endpoints and to estimate the multivariable-adjusted differences in time-to-event efficacy outcomes between the groups of patients with histologically different cancer types. We adjusted the analyses of the TTR, DOR, and PFS for the eight previously mentioned covariates and analysis of the OS additionally for therapy after the discontinuation of nivolumab and the time-dependent covariate number of cycles. Before the analysis, we tested the proportional hazard assumption by visually inspecting the parallelism of log-log survival plots drawn separately for patients with squamous and nonsquamous NSCLC, both of which were unadjusted and adjusted for all planned covariates, by comparing the closeness of Kaplan–Meier observed survival curves and the Cox regression predicted curves for the same endpoint and by assessing the consistency of the log HR overtime via a test of the nonzero slope of the generalized linear regression of the scaled Schoenfeld residuals on row time, as well as on the log time. To facilitate the interpretation of time-to-response, in addition to the median time to response, we calculated the probability that a randomly selected patient with squamous NSCLC would reach the response before a randomly selected patient with nonsquamous NSCLC. We calculated this probability as *p*=HR/(1+HR), where HR is a hazard ratio for the response. We analyzed the ORR and DCR using binary logistic regression. We performed the primary analysis in the intention-to-treat population, that is, on the total sample of patients who received at least one dose of nivolumab. We did not specify the missing data for each variable in text, but we presented denominators for all percentages and declare missing data below the tables. In all analyses, we used the numeric variables in their original forms and additionally presented the categorized age at nivolumab initiation and the time from diagnosis to introduction of nivolumab in years only for the descriptive purposes. Before the analysis, we combined ECOG status 1 and ECOG status 2 because there were only 4 patients with ECOG status 2. We set two-tailed statistical significance at *p* < 0.05 and calculated all confidence intervals (CIs) at the 95% level. We controlled the false positive rate using the Benjamini–Hochberg procedure with the false discovery rate set in advance at FDR<10%. We performed the statistical data analysis using StataCorp 2019 (Stata Statistical Software: release 16. College Station, TX: StataCorp LLC).

## 3. Results

### 3.1. Patients Characteristics

We assessed for eligibility of all patients who were enrolled in the Named Patient Program (NPP) in Croatia, Hungary, and Malta from April 2015 to December 2016. Finally, we enrolled 243 patients with advanced NSCLC from five hospitals. We excluded four patients because they died before the commencement of nivolumab treatment. We analyzed the data on 239 patients who received at least one dose of nivolumab during the enrollment period. The median (IQR) age of the patient at the introduction of nivolumab was 62 (57–68) years, with 39 (16.3%) patients younger than 55 years and 16 (6.7%) patients ≥75 years; the overall age range was from 34 to 79 years. A total of 81/239 (33.9%) patients had squamous NSCLC and 158/239 (66.1%) patients had nonsquamous NSCLC; 78/239 (32.6%) were women (Table 1). In patients with nonsquamous NSCLC, 155/158 (98.1%) had adenocarcinoma, 1 (0.6%) had large cell NSCLC, and 2 (1.3%) had NOS. Only 4/239 (1.7%) patients had an ECOG performance status of 2 or higher at the beginning of the treatment. The most frequent site of metastasis was the mediastinal lymph nodes (81%), followed by the lung (78.4%), bones (31.9%), liver (16.7%), and brain (16%). Patients received a median (IQR) of 12 (4–28) doses of nivolumab, and the median (IQR) follow-up duration from the introduction of nivolumab to the last visit or death was 13 (4–24) months, with the overall range from 0 to 44 months. Before the introduction of nivolumab, 218/229 (95.2%) patients were treated with chemotherapy for metastatic disease and 60/232 (25.9%) were treated with tyrosine kinase inhibitors. Treatment with nivolumab was discontinued for any reason in 96/232 (41.4%) patients. At the end of follow-up, 37/239 (15.5%) patients were alive without disease progression (censored cases).

### 3.2. Toxicity

Treatment was discontinued because of toxicity in 27/232 (11.6%; 95% CI 7.8% to 16.5%) patients (Table 2), and we did not observe any treatment-related deaths. In patients with squamous cell NSCLC, treatment was discontinued because of toxicity in 12/80 patients (15.0%; 95% CI 7.1% to 22.8%), and in patients with nonsquamous cell NSCLC, treatment was discontinued in 15/152 patients (9.9%; 95% CI 5.2% to 14.6%). The difference in the number of patients whose treatment was discontinued because of toxicity was not significant between squamous and nonsquamous NSCLC (Fisher's exact test, *p*=0.283; FDR > 10%). Treatment-related immune-mediated adverse events were experienced by 44/239 patients (18.4%; 95% CI 13.7% to 23.9%), and 10/239 patients (4.2%) experienced grade III or IV immune-mediated adverse events. Serious adverse events (SAE) (pneumonitis, myasthenia gravis, myositis, arthritis, hepatitis, diabetes mellitus, and cardiotoxicity) were experienced by 16/239 (6.7%) patients. Median (IKR) time from the introduction of nivolumab to the treatment discontinuation because of SAE was 4.4 (1.6–10.7) months. Out of patients with SAE, 3/16 (18.8%) were rechallenged with nivolumab.

### 3.3. Efficacy

**Figure 1:**
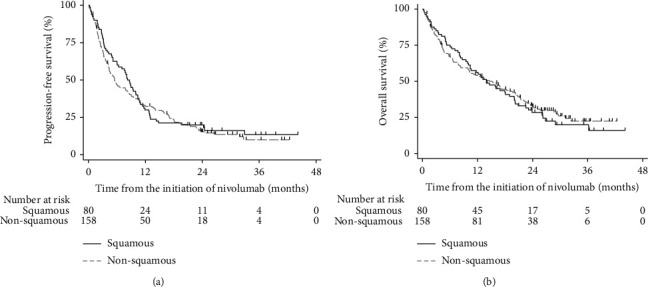
Kaplan–Meier curves of the progression-free and overall survival.

The ORR, including patients who achieved a complete or partial response, was 48/229 (21.0%; 95% CI 15.9% to 26.8%) after a median of 3.2 (95% CI 2.4–5.0) months (Table 2). The DCR, defined as the ORR and stable disease as the best response, was 153/229 (66.8%; 95% CI 60.7% to 72.9%). The median PFS was 6.4 (95% CI 5.2 to 8.6) months, and the median OS was 14.1 (10.6 to 18.0) months (Figure 1). The difference in ORR between the patients with squamous and nonsquamous NSCLC was not significant in the bivariate, unadjusted (OR = 1.12; 95% CI 0.58 to 2.17; *p*=0.729; FDR > 10%) nor in the multivariable analysis adjusted for the eight planned covariates (adjusted OR = 1.38; 95% CI 0.56 to 3.44; *p*=0.485; FDR > 10%). The proportional hazard assumption was satisfied for all four time-to-event endpoints. In the bivariate analysis, we observed a significant and clinically relevant difference between the patients with squamous and nonsquamous NSCLC in the TTR (log-rank test, Χ^2^ = 10.9; *p*=0.001; FDR < 10%) (Table 2). This difference remained significant after adjustment for the eight planned covariates. Patients with squamous NSCLC had a 73% (95% CI 56% to 84%) higher chance for faster treatment response than the patients with nonsquamous NSCLC (adjusted HR = 2.64; 95% CI 1.28 to 5.43). Their median time to response was 2.2 months (95% CI 2.0 to 3.2) compared to the 5.1 months (95% CI 2.6 to 7.0) in the other patient group. However, the duration of response was not significantly different (log-rank test, Χ^2^ = 1.3; *p*=0.247; FDR > 10% in the bivariate analysis, and adjusted HR = 0.70; 95% CI 0.24 to 2.08; *p*=0.527; FDR > 10% in the multivariable, adjusted analysis). PFS was not significantly different in the unadjusted analysis (log-rank test, Χ^2^ = 0.5; *p*=0.466; FDR > 10%), but it was different after the adjustment by multivariable Cox regression (adjusted HR = 0.66; 95% CI 0.48 to 0.93; *p*=0.016; FDR < 10%). Therefore, the patients with squamous NSCLC had a 40% (95% CI 32% to 48%) higher chance for longer PFS than the patients with nonsquamous NSCLC. The difference in PFS was 2.7 months in favor of patients with squamous NSCLC. However, when in the exploratory, subgroup analysis, we adjusted the PFS for the eight planned covariates, and for particular metastatic sites, the PFS was not significantly different between patients with squamous and nonsquamous NSCLC. The OS was not significantly different between these two patient groups in the bivariate (log-rank test, Χ^2^ = 0.16; *p*=0.692; FDR > 10%) nor in the multivariable, adjusted analysis (adjusted HR = 0.86; 95% CI 0.61 to 1.23; *p*=0.016; FDR > 10%). In the exploratory, subgroup multivariable analysis (with the FDR < 10%), we observed a significant independent and unfavorable association of ECOG performance status and the total number of metastatic sites with the PFS and a favorable association of total number of nivolumab cycles and therapy after the nivolumab discontinuation with OS. The variable time from diagnosis to the introduction of nivolumab had *p*=0.021 but an unacceptable FDR > 10%.

## 4. Discussion

Approval of nivolumab use for patients with advanced NSCLC was based on two phase 3 trials (CheckMate 017 and CheckMate 057) [8, 9]. In CheckMate 017, squamous NSCLC patients treated with nivolumab were observed to have higher RRs (20% vs. 9%) and longer durations of response (25.2 vs. 8.4 months) and median OSs (OS 9.2 vs. 6.0 months; HR: 0.62, 95% CI, 0.47–0.80) than patients treated with docetaxel. The median PFS was 3.5 months with nivolumab versus 2.8 months with docetaxel (HR 0.62; 95% CI, 0.47–0.81; *p* < 0.001). The nonsquamous population was studied in the CheckMate 057 trial, and patients treated with nivolumab also had higher RRs (19% vs. 12%) and longer durations of response (17 vs. 5.6 months) and median OSs (OS 12.2 vs. 9.5 months; HR: 0.75, 95% CI, 0.63–0.91) than those treated with docetaxel. Long-term follow-up of these clinical trial results showed that a fraction of the NSCLC patients continued to sustain a durable response [10, 14, 15].

In our study, the ORR we observed in patients with squamous NSCLC was 22% and that in nonsquamous patients was 20%, with a median PFS of 6.4 months and a median OS of 14 months. Furthermore, our results indicated a faster response and longer PFS in patients with squamous than nonsquamous NSCLC, but we did not observe significant differences in DOR or in OS. Our study of real-world data indicated the acceptable safety and clinically relevant efficacy of nivolumab in previously treated advanced NSCLC patients in the South East European countries Croatia, Malta, and Hungary, as well as good consistency with the randomized clinical trials results.

The ORRs we observed in patients with squamous NSCLC (22%) and nonsquamous NSCLC (20%) were somewhat higher than those observed in the comparable Italian study (18% in both histological types) performed on 371 patients with squamous and 1,588 with nonsquamous NSCLC during 2015 at 153 institutions [16, 17]. The DCRs were even higher in our study (73% in squamous and 64% in nonsquamous) than in the Italian study (47% in squamous and 44% in nonsquamous), meaning that the prevalence of stable disease was markedly higher in our study, while the complete and partial responses were almost the same as in the Italian sample. Furthermore, PFS was longer in our study (8.2 months in squamous and 5.5 in nonsquamous) than in the Italian one (4.2 in squamous and 3.0 in nonsquamous), as was OS (13.9 months in squamous and 15.2 in nonsquamous in our study vs. 7.9 in squamous and 11.3 in nonsquamous in the Italian study). These differences may at least be partially explained by the differences between the targeted populations in our and in the Italian study and by the differences in the interventions between the two studies. The median age of patients with squamous NSCLC was five years lower in our study and of patients with nonsquamous NSCLC was six years lower in our study than in the Italian study; in addition, the patients in our study had less CNS metastasis (5% vs. 12% in squamous and 23% vs. 26% in nonsquamous) and better ECOG performance status at the introduction of nivolumab than the patients in the Italian study. In the Italian study, squamous and nonsquamous patients received a median of six and seven doses of nivolumab, respectively, while medians of 13 and 11 doses were administered in our study. Despite these differences, the two real-world studies found very similar efficacy results. The discontinuation rate in the Italian study was lower (5%) than that in our study (12%), which again may at least partially be explained by the 36% to 50% higher doses that were administered to patients with squamous and patients with nonsquamous NSCLC in our study, respectively. The existence of liver or brain metastases was seen in 16% (squamous NSCLC) and 16.7% (nonsquamous NSCLC) of our patients at the introduction of nivolumab treatment. Patients with advanced NSCLC who have liver or brain metastases and are receiving chemotherapy or tyrosine kinase inhibitors are known to have poorer prognoses than those with other metastases [18]. One retrospective study showed a poorer PFS in patients with liver metastases who were treated with nivolumab [19], while another found that nivolumab was effective against brain metastasis [20]. In our study, we did not find differences in PFS or OS regarding liver and brain metastases.

The first limitation of our study is closely related to its main strength, namely, the lack of a randomized control group not treated with nivolumab. Although the key goal of this real-world observational study was to assess the safety and efficacy in a broader population from real clinical settings, the lack of a randomized control group increased the risk of numerous unmeasured confounding effects and certainly lowered the interval validity of our findings. The second limitation is inherent to this formal study design. We did not have control over the validity, reliability, and precision of the data originally entered into the electronic medical records, nor did we have a chance to check the quality of these entries. The main strength of our study was its setting, namely, the natural, real-world, and everyday clinical setting, which covered the population of patients who were actually treated.

Data were missing for 1 patient for the stage; 2 for the EGFR and ALK; 4 for the treatment duration; 5 for the brain status; 7 for the tyrosine kinase inhibitors use, mediastinal lymph nodes, and therapy after nivolumab; 8 for the lung, liver status, and the number of cycles; 10 for the chemotherapy of metastatic disease, bone status, pleural effusion status, pleural metastasis status, and abdominal lymph nodes status; and 15 for the number of metastatic sites.

## 5. Conclusion

The safety and efficacy of nivolumab in previously treated patients with advanced NSCLC in clinics in South East Europe are consistent with those found in randomized clinical trials and comparable to the results from the real-world experience in other countries.

## Figures and Tables

**Table 1:** Characteristics of the patients and treatment.

	All patients (*n* = 239)	Tumor histology	
		Squamous (*n* = 81)	Nonsquamous (*n* = 158)
Demographic characteristics	61 (56–66)	62 (58–66)	60 (55–66)
Age at nivolumab initiation (years), median (IQR)	62 (57–68)	64 (60–67)	62 (57–68)

*Sex*			
Female	78 (32.6)	17 (21.0)	61 (38.6)
Male	161 (67.4)	64 (79.0)	97 (61.4)

*Clinical characteristics*			
Stage at introduction of nivolumab			
III (unresectable)	10 (4.2)	4 (5.0)	6 (3.8)
IV	228 (95.8)	76 (95.0)	152 (96.2)

*Treatment of metastatic disease*			
Chemotherapy	218 (95.2)	74 (94.9)	144 (95.4)
Tyrosine kinase inhibitors	60 (25.9)	11 (13.9)	49 (32.0)

*Metastatic sites*			
Mediastinal lymph nodes	188 (81.0)	68 (85.0)	120 (78.9)
Lung	181 (78.4)	67 (85.9)	114 (74.5)
Bone	73 (31.9)	18 (23.1)	55 (36.4)
Pleural effusion	60 (26.2)	23 (28.7)	37 (24.8)
Other sites	44 (18.4)	10 (12.3)	34 (21.5)
Brain	39 (16.7)	4 (4.9)	35 (22.9)
Liver	37 (16.0)	13 (16.5)	24 (15.8)
Pleural metastasis	17 (7.4)	2 (2.5)	15 (10.0)
Abdominal lymph nodes	17 (7.4)	5 (6.3)	12 (8.0)
Number of metastatic sites, median (IQR)	3 (2–4)	2 (2–3)	3 (2–4)

*ECOG performance status*			
0	125 (52.3)	43 (53.1)	82 (51.9)
1	110 (46.0)	37 (45.7)	73 (46.2)
≥2	4 (1.7)	1 (1.2)	3 (1.9)

EGFR status positive (among those tested)	15/145 (10.3)	0/5 (0.0)	15/140 (10.7)
ALK rearrangements present (among those tested)	2/116 (1.7)	0/2 (0.0)	2/114 (1.8)
Time from diagnosis to introduction of nivolumab (months), median (IQR)	18 (11–30)	16 (11–26)	19 (11–31)
Number of cycles, median (IQR)	12 (4–28)	13 (5–25)	11 (4–29)
Treatment duration (months), median (IQR)	5 (2–12)	6 (2–11)	5 (2–15)
Therapy after nivolumab	83 (35.8)	29 (35.8)	54 (35.8)

Data are presented as the number (percentage) of patients if not stated otherwise. Abbreviations: IQR = interquartile range; EGFR = epidermal growth factor receptor; ALK = anaplastic lymphoma kinase.

**Table 2:** Safety and efficacy of nivolumab.

	All patients (*n* = 239)	Tumor histology	
		Squamous (*n* = 81)	Nonsquamous (*n* = 158)
*Safety endpoints*			
Treatment discontinuation because of toxicity	27 (11.6)	12 (15.0)	15 (9.9)
Treatment-related adverse events			
Any grade	59 (24.7)	20 (24.7)	39 (24.7)
Grades III-IV	12 (5.0)	4 (4.9)	8 (5.1)

*Treatment-related, immune-mediated adverse events*			
Any grade	44(18.4)	16 (19.8)	28 (17.7)
Grades III-IV	10 (4.2)	4 (4.9)	6 (3.8)

*All adverse events*			
Dermatitis, rash, and pruritus	31 (13.0)	6 (7.4)	25 (15.8)
Hypothyroidism	10 (4.2)	4 (4.9)	6 (3.8)
Fatigue	9 (3.8)		9 (5.7)
Diarrhea	8 (3.3)	4 (4.9)	4 (2.5)
Hepatotoxicity	6 (2.5)	2 (2.5)	4 (2.5)
Pneumonitis	5 (2.1)	3 (3.7)	2 (1.3)
Anemia	5 (2.1)	3 (3.7)	2 (1.3)
Neuropathy	4 (1.7)		4 (2.5)
Fever, infection	3 (1.3)	2 (2.5)	1 (0.6)
Other, each <1%^∗^	13 (5.4)	6 (7.4)	7 (4.4)

*Efficacy endpoints*			

*Best objective response, n (%)*			
Complete response (CR)	5 (2.2)	1 (1.2)	4 (2.7)
Partial response (PR)	43 (18.8)	17 (21.0)	26 (17.6)
Stable disease (SD)	105 (45.9)	41 (50.6)	64 (43.2)
Progressive disease (PD)	35 (15.3)	10 (12.3)	25 (16.9)
Could not be determined	41 (17.9)	12 (14.8)	29 (19.6)
Objective response rate, *n* (%)	48 (21.0)	18 (22.2)	30 (20.3)
Disease control rate, *n* (%)	153 (66.8)	59 (72.8)	94 (63.5)
TTR (months), median (95% CI)	3.2 (2.4–5.0)	2.2 (2.0–3.2)	5.1 (2.6–7.0)
Unadjusted HR (95% CI)		3.05 (1.53–6.07)	1.00 (referent)
Adjusted HR (95% CI)^†^		2.64 (1.28–5.43)	1.00 (referent)
DOR (months), median (95% CI)	19.1 (12.9-†)	31.0 (9.6-‡)	17.2 (12.2-‡)
Unadjusted HR (95% CI)		0.61 (0.26–1.42)	1.00 (referent)
Adjusted HR (95% CI)^†^		0.70 (0.24–2.08)	1.00 (referent)
PFS (months), median (95% CI)	6.4(5.2–8.6)	8.2 (6.0–10.4)	5.5 (4.4–8.3)
Unadjusted HR (95% CI)		0.90 (0.67–1.20)	1.00 (referent)
Adjusted HR (95% CI)^†^		0.66 (0.47–0.93)	1.00 (referent)
OS (months), median (95% CI)	14.1 (10.6–18.0)	13.9 (10.0–20.0)	15.2 (10.0–20.3)
Unadjusted HR (95% CI)		1.07 (0.78–1.46)	1.00 (referent)
Adjusted HR (95% CI)^†^		0.86 (0.61–1.23)	1.00 (referent)

Abbreviations: CI = confidence interval; TTR = time to response; DOR = duration of response, PFS = progression-free survival; OS = overall survival. ^*∗*^Other adverse events observed in <1% of patients each were arthritis, migrating arthralgia, myositis, cardiotoxicity, herpes zoster ophthalmicus, colitis, thrombocytopenia, pain, myalgia, encephalopathy, and nausea. ^†^Analysis of TTR, DOR and PFS was adjusted for age at the introduction of nivolumab, sex, previous treatment of metastatic disease with tyrosine kinase inhibitors, number of metastatic sites, ECOG performance status at introduction of nivolumab, time from diagnosis to introduction of nivolumab (years), and, as the time-dependent covariate, concomitant radiotherapy; analysis of OS was additionally adjusted for therapy after the discontinuation of nivolumab and the time-dependent covariate number of cycles. ^‡^Statistic could not be estimated. Data were missing for 3 patients for time to response and duration of response; 7 patients for treatment discontinuation; and 10 patients for the best response.

## Data Availability

The data used to support the findings of this study are available from the corresponding author upon request.
